# Supplementary material for: dCas9-BE3 and dCas12a-BE3 Systems Mediated Base Editing in Kiwifruit Canker Causal Agent Pseudomonas syringae pv. actinidiae
Source: Int J Mol Sci. 2023 Feb 27;24(5):4597. doi: 10.3390/ijms24054597 (PMC10003707; doi:10.3390/ijms24054597)
Supplement: Supplementary file 1 [file ijms-24-04597-s001.zip › ijms-2213190-supplementary.pdf]

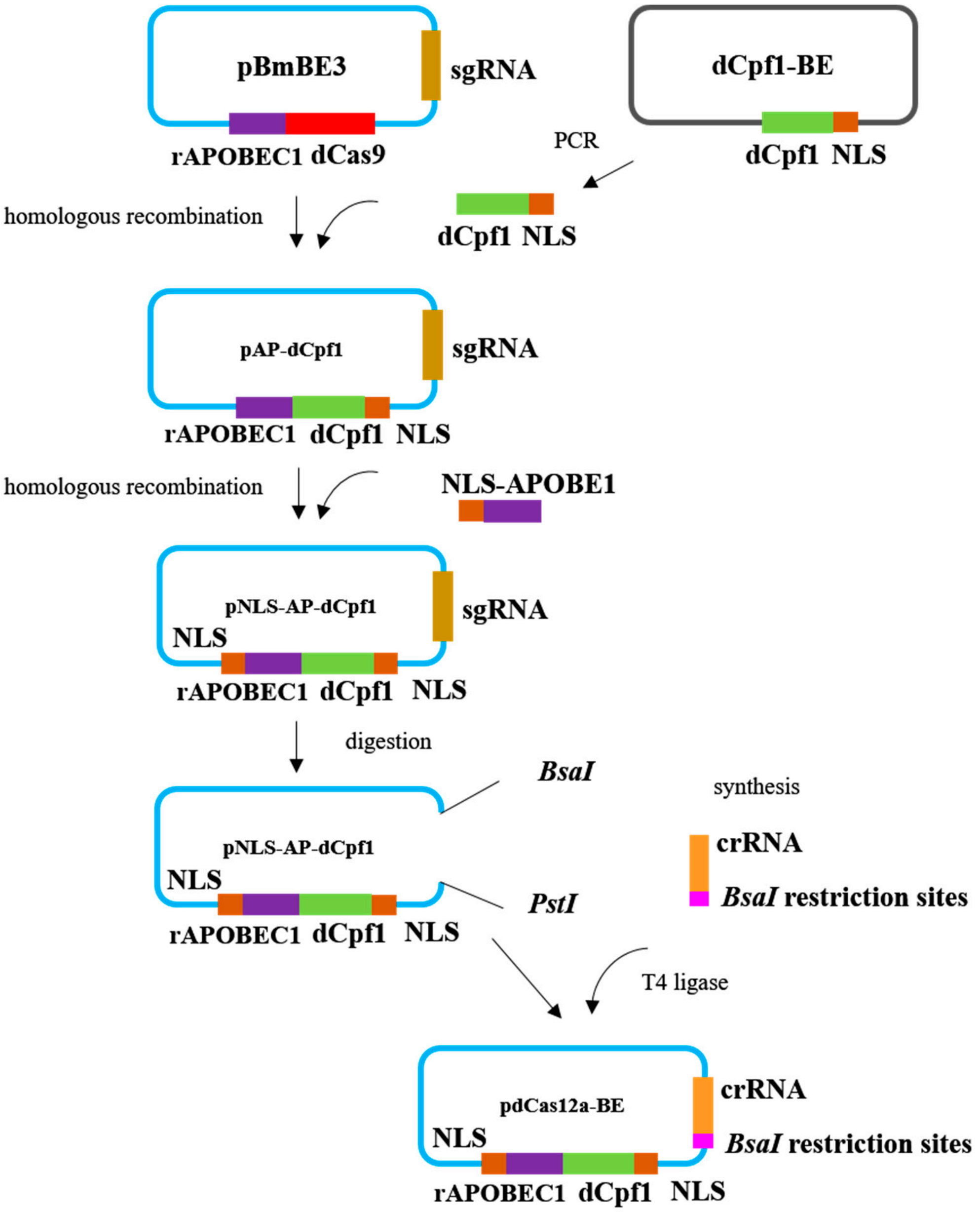

Supplementary Figure S1. Construction flow chart of pdCas12a-BE vector

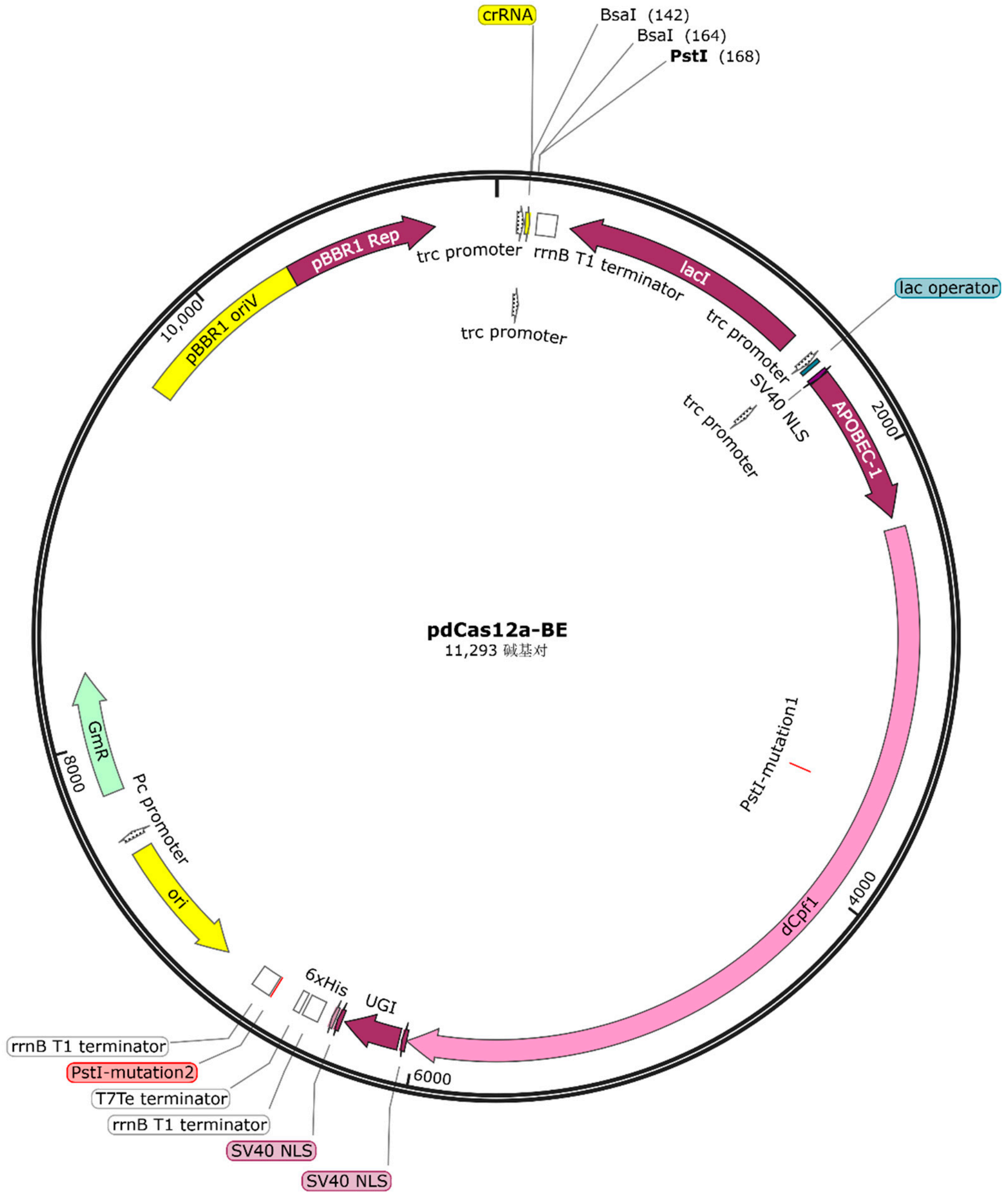

Supplementary Figure S2. Scheme of pdCas12a-BE vector

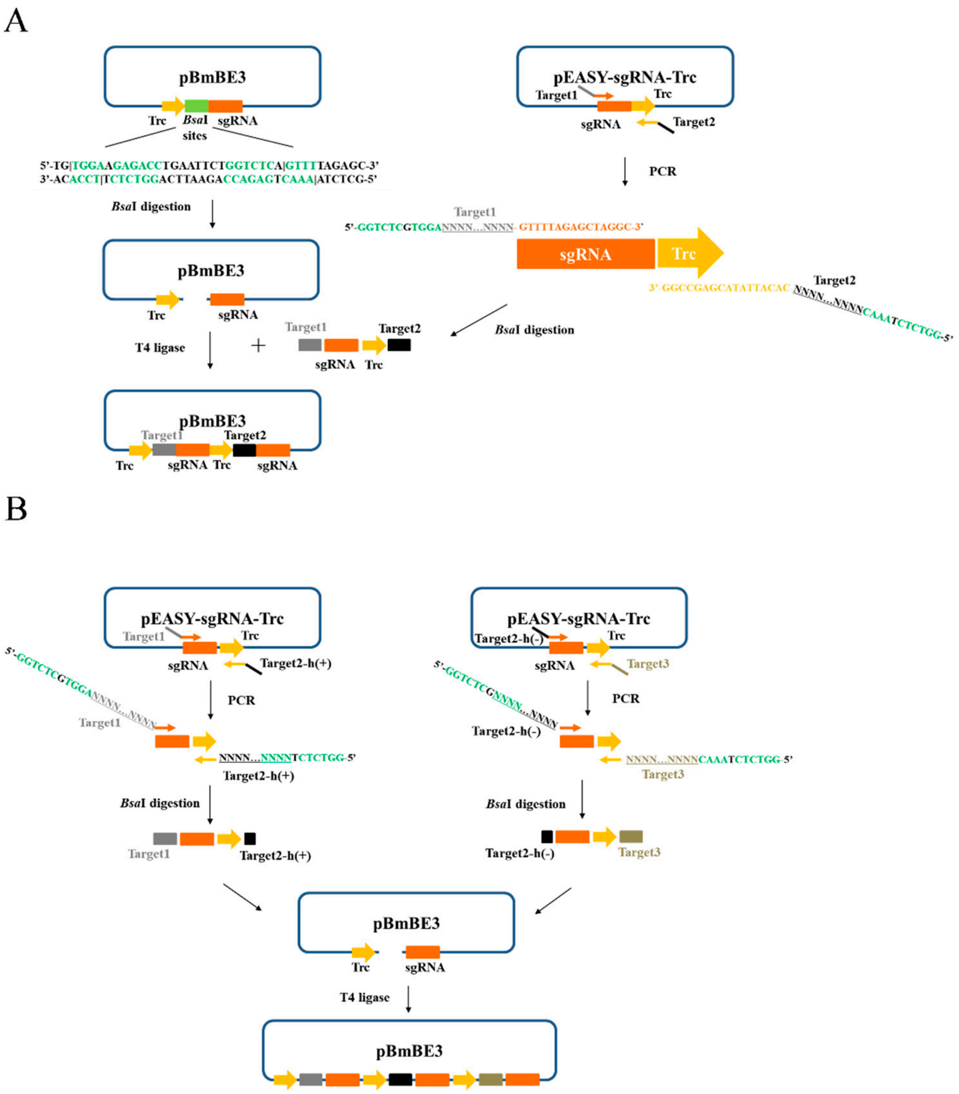

Supplementary Figure S3 Construction flow chart of multi-site knockout vectors. A. The construction flow chart of double sites knockout vector. B. The construction flow chart do triple sites knockout vector.

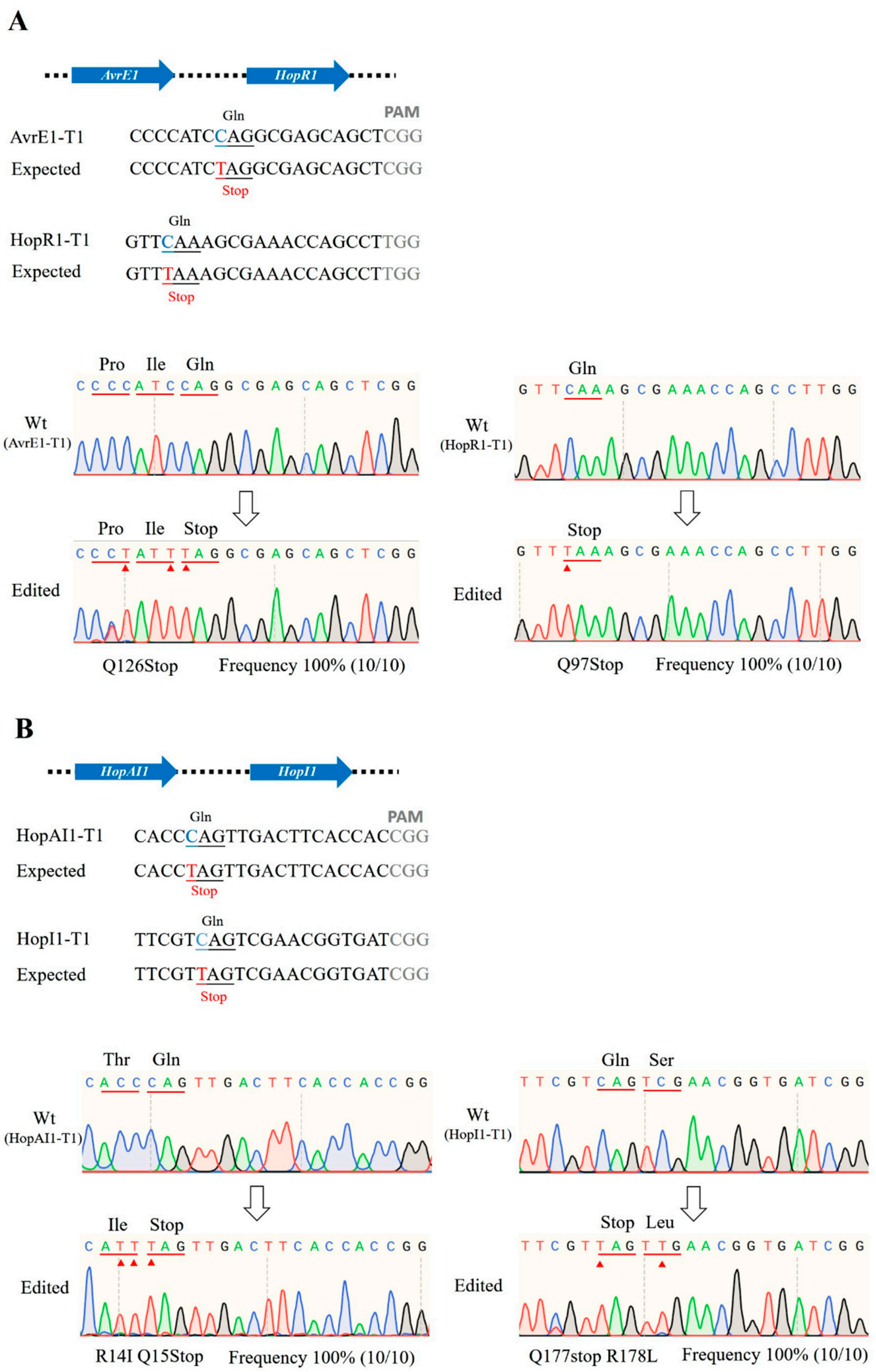

Supplementary Figure S4. Two sites knockout by using dCas9-BE3 system in *Psa*.

A. The result of *AvrE1* and *HopR1* gene knockout by using dCas9-BE3 system in *Psa*.

B. The result of *HopAI1* and *HopI1* gene knockout by using dCas9-BE3 system in *Psa*.

```
PSA.AH.01_HopF2 MGNVCGTSGSHHVYSPPVSPRHVSGSSTPVHNVAGQALTSVYQLSDEAREDFLSRHDPMQ
PtoDC3000_HopF2 IGNICGTSGSRHVYSPSHTQRITSAPSTSTH-VGGDTLTSIHQLSHSQREQFLNMHDPMR
Pph1449b_HopF1  MGNICSSGGVSRTYSPPTSPVHGSGVSSPSRFVGQYTLTSIHQLSSEERENFLDAHDPMR
                :**:*.:.*  :.***. :    *. *:. : *.  :***::*** . **:**. ****:

PSA.AH.01_HopF2 KLGLHSETALYRTTDKTYLRGGKLAGNPESCARIGLHEELAPNPYAQHYGIPEGDSRAYR
PtoDC3000_HopF2 VMGLDHDTELFRTTDSRYIKNDKLAGNPQSMASILMHEELRPNRFASHTGAQPHEARAYV
Pph1449b_HopF1  VYDLNSETSVYRTTPREYVRNGYATGNPNSGATIALHEELQESPYAQHIGARPDQADAYR
                  .*.  :* ::***   *::..   :***:* * * :****  .  :*.* *    :: **

PSA.AH.01_HopF2 PREMRASDLRDPSLNVMVGSEARDAVRGY--ASGNHVAVKMRLGDFLEKGGKVYSDVSAV
PtoDC3000_HopF2 PKRIKATDLGVPSLNVMTGSLARDGIRAYDHMSDNQVSVKMRLGDFLERGGKVYADASSV
Pph1449b_HopF1  PRTAHASSLNTPSLNVMAGQGALSALSNY--ARSDHVTTEMKLGDFLDQGGKVYSDNSAM
                *:   :*:.*  ******.*.  * ..:  *    .::*:.:*:*****::*****:* *::

PSA.AH.01_HopF2 ASNGDTASALIVTLPKGRKVPAQVVDD
PtoDC3000_HopF2 ADDGETSQALIVTLPKGQKVPVERV--
Pph1449b_HopF1  SAGGDRVEALIVTLPKGRKVPVNILD-
                :  .*:   .*********:***.:  :
```

Supplementary Figure S5. Alignment of hopF2 protein sequences.

```
DC3000_HopAO1    MNPLQPIQHSITNSQM----SGGQQLEAEGSQAHNSYSHPDRISLSQLSQSAHLALDHLS
PSA_HopAO2       MQ-----NHVITSRGVIPLSRGGSASDLEPAYA-------------QLTPEAY-------
                 *:     :* **.  :     **.  : * : *             **: .*:

DC3000_HopAO1    TQPNTDHQRVASLVRNAVQDGKFQLQSSNDTQVTYKTSVCPPANADTMGAAHLINNELTV
PSA_HopAO2       --------------RHAVT--KFK------------------------------------
                               *:**   **:

DC3000_HopAO1    QARLNDQLEYDIVSAHLYGPSEAISIDASSPPSANDLASSGLSERTHLGMNRVLLRYAVP
PSA_HopAO2       ---------------------QALT---------------------------------TP
                                      :*::                                 .*

DC3000_HopAO1    PRETEDQCVMVIDKMPPPKHGKMSFFRTTNDLSKLPLGMETGGLSDLKLAGCERISSVEQ
PSA_HopAO2       ERPTGEP-VFLYDRTP----GELENFRSSDSFI-LPHHLNQKGWDTLHISGSASIASLEQ
                  * * :  *:: *: *    *::. **:::.:  **  ::  * . *:::*.  *:*:**

DC3000_HopAO1    VKSIRAALGGGPLTVLDLREESHAIVNGLPITLRGPMDWANAGLSQVDGAARESAMITEL
PSA_HopAO2       VQRLHPT-PESPVVVLDVREESHAIVGGYPCTWRLGNNWANVGKSRNAVIADEQSRIAAL
                 *: ::.:   .*:.***:********.* * * *   :***.* *:    * *.: *: *

DC3000_HopAO1    KRTKSLTLVDANYVKGKKSNPQTTELKNLNVRSEREVVTEAGATYRRVAITDHNRPSPEA
PSA_HopAO2       KQQPTVEIIHRKDAKHGLENPRKVVLKNPDISSEEDLVKSTGAGYLRLMVTDHMGPRSED
                 *:  :: ::. : .*   .**:.. *** :: **.::*..:** * *: :***  * .*

DC3000_HopAO1    TDELVDIMRHCLQANESLVVHCNGGRGRTTTAMIMVDMLKNARNHSAETLITRM------
PSA_HopAO2       ID-LFLAMERALPEHGRVHIHCGVGQGRTGIFIAMHDMLKNAHHVSFHDLIERQLAFNPG
                  * *.  *.:.*  :  : :**. *:***   : * ******:: * . ** *

DC3000_HopAO1    AKLSYDYNMTDLGSISALKRPFLEDRLKFLQAFHDYARNNPSGLSLNWTQWRAKIALE--
PSA_HopAO2       RALDFNKDVTHEG-----RANLRNDRLEFISLFYEYAKQNPKGAPRSWSEWLADANTPSQ
                   *.:: ::*. *     :  : :***:*:. *::**::**.* . .*::* *.

DC3000_HopAO1    ---
PSA_HopAO2       QRF
```

Supplementary Figure S6. Alignment of hopAO2 protein sequences.

Supplementary Table S1. Statistical table of orthogonal experiment results for the preparation of *Psa* chemically competent cells

Number	Factors			Number of *Psa*
	Concentrations of $CaCl_2$ (mmol/L)	Heat shock time(min)	Recovery time(h)	
1	1(25)	1(1.5)	1(1.0)	72.33
2	1	2(3.0)	2(2.0)	452.33
3	1	3(4.5)	3(3.0)	480.67
4	1	4(6.0)	4(4.0)	569.33
5	2(50)	1	2	950.00
6	2	2	1	533.33
7	2	3	4	660.00
8	2	4	3	662.00
9	3(75)	1	3	207.67
10	3	2	4	672.67
11	3	3	1	175.67
12	3	4	2	414.67
13	4(100)	1	4	394.33
14	4	2	3	262.00
15	4	3	2	270.67
16	4	4	1	203.33
Average 1	393.67	406.08	246.17	
Average 2	701.33	480.08	521.92	
Average 3	367.67	396.75	403.08	
Average 4	282.58	462.33	574.08	

When the concentrations of $CaCl_2$ was 50mmol/L, the number of *Psa* was 701.33 which was the optimal condition. Similarly, the best of heat shock time and recovery time was 3min and 4h.

Supplemental Table S2.
Single clone sequencing of the sgRNA library in bacteria

	No. of tested clones	No. of clones with correct sgRNAs*	No. of clones with incorrect sgRNAs*	No. of clones with no sgRNA
DH5α	365	307 (84.1%)	55 (15.1%)	3 (0.8%)

*There were 298 different sgRNAs detected in the 307 bacteria clones

Supplementary Table S3.
Statistics of Psa genome knockout target gene numbers

Psa.strain	Total number of genes	Having dCas9 targets	Having dCas12a targets	Having no dCas9 targets	Having only 1 dCas9 target
PAH.AH.01	6079	5728	3287	351	374
ICMP9853	6074	5822	3362	252	366
ICMP18708	5703	5514	3246	189	294
P220	5787	5581	3332	206	309
M228	5884	5670	3423	214	313

Supplementary Table S4.
Statistics of *Psa* genome knockout target gene numbers with no and only 1 dCas9 targets

Psa.strain	Total number of genes	Having no dCas9 targets		Having only 1 dCas9 target	
		Having dCpf1 targets	Having no dCpf1 targets	Having dCpf1 targets	Having no dCpf1 targets
PAH.AH.01	6079	61	290	79	295
ICMP9853	6074	48	204	101	265
ICMP18708	5703	33	156	76	218
P220	5787	34	172	79	230
M228	5884	40	174	85	228

Supplementary Table S5
Primers and their applications.

Name	Sequence	Use
PSA-F1	TTTTGCTTTGCACACCCGATTTT	Molecular identification of *Psa* strains
PSA-R1	CACGCACCCTTCAATCAGGATG	
27F	AGAGTTTGATCCTGGCTCAG	
1492R	GGTTACCTTGTTACGACTT	
M13F	GTAAAACGACGGCCAGT	Construction of pdCpf1-BE3 vector
M13R	CAGGAAACAGCTATGAC	
BmBE3-PCR-F	TCTGGTGGTTCTACTAATCTGTCAG	
BmBE3-PCR-R	ACTTTCGGGTGTGGCGGACT	
dCpf1-F	agtccgccacacccgaaagtATGTCAAAGCTGGAGAAATT	
dCpf1-NLS-R	gactttcctcttcttcttgggTGATCCATGCTTCACTGAGGTCTG	
dCpf1-NLS-SGGS-R	agattagtagaaccaccagaGACTTTCCTCTTCTTCTTGGGTG	
dCpf1-jiance-F	TTCTTGAACTGTACTGCA	
dCpf1-jiance-R	TCGGTGCTCTCGTCGTAG	
NLS-AP-F	catATGCCCAAGAAGAAGAGGAAAGTCATGAGCTCAGAGACTGGCC	
TY-NLS-AP-F	ctaaggaagctaaaatgcatATGCCCAAGAAGAAGAGGAAAGT	
TY-NLS-AP-R	ccgggagtctcgctgccgctTTTCAACCCGGTGGCCC	
TY-NLS-AP-AUTIPCR-F	AGCGGCAGCGAGACTCCC	
TY-NLS-AP-AUTIPCR-R	ATGCATTTTAGCTTCCTTAGCTCC	
NLS-AP-jiance-F	TTTGCGCCATTGAGCTCAAT	
NLS-AP-jiance-R	GGACTCTGAGGTCCCGGGAG	
PstI-mutation-F1	TCCGCTCTGAACCTGCAaGAGGCAT	
PstI-mutation-R1	ATGCCTCtTGCAGGTTCAGAGCGGA	
PstI-mutation-F2	ACAGCTGCAaGAGTACGCCGACGCTGACCTGT	
PstI-mutation-R2	CGTACTCtTGCAGCTGTTCGAGAGAGAAGCTA	
crRNA-BsaI-F	TGGAAATTTCTACTAAGTGTAGATCGAGACCCGAGGGTCTCCTGCA	
crRNA-BsaI-R	GGAGACCCTCGGGTCTCGATCTACACTTAGTAGAAATT	
bsaI-crRNA-jiance-F	CGGTCACGGATCCGCGCG	
bsaI-crRNA-jiance-R	GCGCAACGCAATTAAGTG	
flic-target1-f	TGGAGCTCAAAGTACCAACCTGAA	Construction of mutation vector
flic-target1-r	AAACTTCAGGTTGGTACTTTGAGC	

hopQ1-target3-f	TGGATCGCTCTCAACGTGCCGAGA
hopQ1-target3-r	AAACTCTCGGCACGTTGAGAGCGA
avrE1-target1-f	TGGACCCCATCCAGGCGAGCAGCT
avrE1-target1-r	AAACAGCTGCTCGCCTGGATGGGG
hopR1-target1-f	TGGAGTTCAAAGCGAAACCAGCCT
hopR1-target1-r	AAACAGGCTGGTTTCGCTTTGAAC
hrcc-target1-f	TGGAGACCAGCAGTTAGTGGTGGC
hrcc-target1-r	AAACGCCACCACTAACTGCTGGTC
avrB4-target1-f	TGGATATTATCAGCAATCGTTAAT
avrB4-target1-r	AAACATTAACGATTGCTGATAATA
hopI1-target1-f	TGGATTCGTCAGTCGAACGGTGAT
hopI1-target1-r	AAACATCACCGTTCGACTGACGAA
hopI1-target2-f	TGGAGCGCGTCCGCAAACGCCTCC
hopI1-target2-r	AAACGGAGGCGTTTGCGGACGCGC
hopQ1-target2-f	TGGAGGGCAAGCAATTGCAGGCTG
hopQ1-target2-r	AAACCAGCCTGCAATTGCTTGCCC
hopQ1-target1-f	TGGAACAAATATCACGTACCCCAT
hopQ1-target1-r	AAACATGGGGTACGTGATATTTGT
hopAI1-target1-f	TGGACACCCAGTTGACTTCACCAC
hopAI1-target1-r	AAACGTGGTGAAGTCAACTGGGTG
hopZ3-target1-f	TGGATACTCAGCAGGCTGAGGGTT
hopZ3-target1-r	AAACAACCCTCAGCCTGCTGAGTA
hopAO2-target1-f	TGGACAAACAAGCGCTGACTACAC
hopAO2-target1-r	AAACGTGTAGTCAGCGCTTGTTTG
hopM1-target1-f	TGGAATTCCAGGCCGAGCAGGCGA
hopM1-target1-r	AAACTCGCCTGCTCGGCCTGGAAT
hopF2-target1-f	TGGAAGTGTATCAGTTGTCCGACG
hopF2-target1-r	AAACCGTCGGACAACTGATACACT
cpf1-AI1-T2-F	TGGATAATTTCTACTAAGTGTAGATGCCTTGAATCACCGTG CACCTAC
cpf1-AI1-T2-R	AAACGTAGGTGCACGGTGATTCAAGGCATCTACACTTAGT AGAAATTA
cpf1-I1-T1-F	TGGATAATTTCTACTAAGTGTAGATAAGAACCACCCTGATA AAAAACC
cpf1-I1-T1-R	AAACGGTTTTTATCAGGGTGGTTCTTATCTACACTTAGTA GAAATTA
cpf1-Q1-T1-F	TGGATAATTTCTACTAAGTGTAGATTCACTGATCCTAACAA GGACCCG
cpf1-Q1-T1-R	AAACCGGGTCCTTGTTAGGATCAGTGAATCTACACTTAGTA GAAATTA
cpf1-Q1-T2-F	TGGATAATTTCTACTAAGTGTAGATTGCACATCACGGACGT AGTGGCA
cpf1-Q1-T2-R	AAACTGCCACTACGTCCGTGATGTGCAATCTACACTTAGTA GAAATTA

cpf1-FLIC-T1-F	TGGATAATTTCTACTAAGTGTAGATGACCTCGATGACTCGT CTGTCCT
cpf1-FLIC-T1-R	AAACAGGACAGACGAGTCATCGAGGTCATCTACACTTAGT AGAAATTA
cpf1-AI1-T1-F	TGGATAATTTCTACTAAGTGTAGATAAGCTGAACACCCAGT TGACTTC
cpf1-AI1-T1-R	AAACGAAGTCAACTGGGTGTTCAGCTTATCTACACTTAGTA GAAATTA
hopAl1-T1-jiance-f	ATGCCCGTAAGCAAACCC
hopAl1-T1-jiance-r	CTTGCCTATCGCATCAAA
hopQ1-T1-jiance-f	ATGCATCGTCCTATCACC
hopQ1-T1-jiance-r	TCAATCTGGGGCTACCGT
hopI1-T1-jiance-f	ATGATCAACCTCACCCA
hopI1-T1-jiance-r	TCACGCCCTGGCATCAGGTA
FLIC-T1-jiance-f	ATGGCTTTAACAGTAAAC
FLIC-T1-jiance-r	TTACTGAAGCAGTTTCAG
hopZ3-T1-jiance-f	ATGAATATCTCAGGTCTG
hopZ3-T1-jiance-r	TTACGCCCTGACCCTGTT
avrE1-T1-jiance-f	ATGTATAGAGTTTCCGGT
avrE1-T1-jiance-r	TCAGTCAATCACATGCGC
hopAO2-T1-jiance-f	ATGCAGAATCATGTCATT
hopAO2-T1-jiance-r	CTAAAAGCGTTGTTGAGA
hopF2-T1-jiance-f	ATGGGTAATGTTTGTGGT
hopF2-T1-jiance-r	TCAGTCGTCCACTACCTG
hopM1-T1-jiance-f	ATGAGCGACATGAGAATC
hopM1-T1-jiance-r	TTAGCGTCTGCGGCCCTC
avrB4-T1-jiance-f	ATGGGATGTATATCATCG
avrB4-T1-jiance-r	TCAGCGATCAAAAAAGTT
hopR1-T1-jiance-f	ATGGTCAAGGTTACCTCTTCC
hopR1-T1-jiance-r	CAACGGCAGCAAATCCAC
hrcc-T1-jiance-f	ATGCGCAAGGCCTTAATG
hrcc-T1-jiance-r	TCATGGTTTCGCTCCCCG

Supplementary Notes 1

Complete sequence of pdCas12a-BE vector

ACGGTCACGGATCCGCGCGCCAGGGTTTTCCCAGTCACGACCTCGAGAAAAAAGCCAGAC
AAACGTCCGGCTTTTGAATTAAGGCCTCCCTTGACAATTAATCATCCGGCTCGTATAATGTGT
GGAAATTTCTACTAAGTGTAGATCGAGACCCGAGGGTCTCCTGCAGGAGGCATCAAATAAA
ACGAAAGGCTCAGTCGAAAGACTGGGCCTTTCGTTTTATCTGTTGTTTGTCGGTGAACGCT
CTCCTGAGTAGGACAAATGGGTAAAAAAAAACCCCGCTTCGGCGGGGTTTTTTTTTACTA
CACTTAATTGCGTTGCGCTCACTGCCCGCTTTCCAGTCGGGAAACCTGTCGTGCCAGCTGC
ATTAATGAATCGGCCAACGCGCGGGGAGAGGCGGTTTGCGTATTGGGCGCCAGGGTGGTTT
TTCTTTTCACCAGTGAGACGGGCAACAGCTGATTGCCCTTCACCGCCTGGCCCTGAGAGAG
TTGCAGCAAGCGGTCCACGCTGGTTTGCCCCAGCAGGCGAAAATCCTGTTTGATGGTGGTT
AACGGCGGGATATAACATGAGCTGTCTTCGGTATCGTCGTATCCCACTACCGAGATATCCGC
ACCAACGCGCAGCCCGGACTCGGTAATGGCGCGCATTGCGCCCAGCGCCATCTGATCGTTG
GCAACCAGCATCGCAGTGGGAACGATGCCCTCATTCAGCATTTGCATGGTTTGTTGAAAAC
CGGACATGGCACTCCAGTCGCCTTCCCGTTCCGCTATCGGCTGAATTTGATTGCGAGTGAGA
TATTTATGCCAGCCAGCCAGACGCAGACGCGCCGAGACAGAACTTAATGGGCCCGCTAACA
GCGCGATTTGCTGGTGACCCAATGCGACCAGATGCTCCACGCCCAGTCGCGTACCGTCTTC
ATGGGAGAAAATAATACTGTTGATGGGTGTCTGGTCAGAGACATCAAGAAATAACGCCGGA
ACATTAGTGCAGGCAGCTTCCACAGCAATGGCATCCTGGTCATCCAGCGGATAGTTAATGAT
CAGCCCACTGACGCGTTGCGCGAGAAGATTGTGCACCGCCGCTTTACAGGCTTCGACGCCG
CTTCGTTCTACCATCGACACCACCACGCTGGCACCCAGTTGATCGGCGCGAGATTTAATCGC
CGCGACAATTTGCGACGGCGCGTGCAGGGCCAGACTGGAGGTGGCAACGCCAATCAGCAA
CGACTGTTTGCCCGCCAGTTGTTGTGCCACGCGGTTGGGAATGTAATTCAGCTCCGCCATCG
CCGCTTCCACTTTTTCCCGCGTTTTCGCAGAAACGTGGCTGGCCTGGTTCACCACGCGGGA
AACGGTCTGATAAGAGACACCGGCATACTCTGCGACATCGTATAACGTTACTGGTTTGTGCA
TGGTATAACCCTTGGTGTTATAGACGTTCCGGGCGCTATCATGCCATACCGCGAAAGGTTTT
GCGCCATTGAGCTCAATGATACGGCGACCACCGAAATTGACAATTAATCATCCGGCTCGTAT
AATGTGTGGAATTGTGAGCGGATAACAAAGATTTTCAGGAGCTAAGGAAGCTAAAATGCAT
CCCAAGAAGAAGAGGAAAGTCATGAGCTCAGAGACTGGCCCAGTGGCTGTGGACCCCAC
ATTGAGACGGCGGATCGAGCCCCATGAGTTTGAGGTATTCTTCGATCCGAGAGAGCTCCGC
AAGGAAACCTGCCTGCTTTACGAAATTAATTGGGGGGGCCGGCACTCCATTTGGCGACATA
CATCACAGAACACTAACAAGCACGTCGAAGTCAACTTCATCGAGAAGTTCACGACAGAAA
GATATTTCTGTCCGAACACAAGGTGCAGCATTACCTGGTTTCTCAGCTGGAGCCCATGCGGC
GAATGTAGTAGGGCCATCACTGAATTCCTGTCAAGGTATCCCCACGTCACTCTGTTTATTTAC
ATCGCAAGGCTGTACCACCACGCTGACCCCCGCAATCGACAAGGCCTGCGGGATTTGATCT
CTTCAGGTGTGACTATCCAAATTATGACTGAGCAGGAGTCAGGATACTGCTGGAGAAACTT
TGTGAATTATAGCCCGAGTAATGAAGCCCACTGGCCTAGGTATCCCCATCTGTGGGTACGAC
TGTACGTTCTTGAACTGTACTGCATCATACTGGGCCTGCCTCCTTGTCTCAACATTCTGAGA
AGGAAGCAGCCACAGCTGACATTCTTTACCATCGCTCTTCAGTCTTGTCATTACCAGCGACT
GCCCCCACACATTCTCTGGGCCACCGGGTTGAAAAGCGGCAGCGAGACTCCCGGGACCTC
AGAGTCCGCCACACCCGAAAGTATGTCAAAGCTGGAGAAATTCACCAACTGTTATAGCCTG
TCTAAGACCCTGCGCTTCAAGGCAATCCCAGTGGGCAAGACACAAGAGAACATTGACAAC

AAACGGCTCCTGGTGGAGGATGAGAAGAGGGCTGAAGATTACAAGGGCGTTAAGAAGCTG
CTGGATAGGTACTATCTGTCATTCATCAACGATGTCCTCCACAGTATCAAGCTGAAGAATCT
GAACAATTACATTTCTCTGTTCCGGAAGAAGACACGGACCGAGAAGGAGAACAAAGAGCT
GGAGAATCTGGAGATCAACCTGAGGAAAGAAATAGCTAAGGCTTTCAAAGGGAACGAGGG
TTACAAGTCCCTGTTCAAGAAAGACATTATCGAGACTATTCTGCCTGAGTTCCTGGACGATA
AAGATGAGATCGCCCTCGTCAATTCCTTCAATGGGTTTACCACAGCCTTTACCGGCTTCTTC
GACAATAGAGAGAATATGTTCTCTGAAGAGGCCAAATCCACTAGCATCGCCTTTCGCTGCAT
AAACGAGAACCTGACTAGGTACATCAGCAATATGGACATCTTTGAGAAAGTCGATGCCATAT
TCGACAAACATGAGGTGCAGGAGATTAAGGAGAAGATCCTGAACTCAGATTACGATGTCGA
AGATTTCTTCGAGGGAGAGTTCTTCAACTTCGTGCTCACACAAGAGGGCATTGATGTGTAC
AATGCAATCATTGGAGGGTTCGTGACAGAGAGTGGCGAGAAGATAAAGGGCCTGAACGAG
TATATCAACCTCTACAACCAGAAAACCAAGCAGAAACTGCCTAAGTTCAAGCCACTGTACA
AACAAGTGCTCTCAGATAGGGAAAGCCTGAGCTTCTACGGTGAAGGGTATACATCAGATGA
AGAAGTGCTCGAAGTGTTCCGCAACACCCTCAATAAGAACAGTGAAATCTTCTCTTCAATC
AAGAAGCTGGAGAAACTGTTCAAGAATTTCGATGAGTACTCCTCTGCCGGAATCTTTGTGA
AGAATGGCCCTGCAATATCCACTATTAGCAAAGACATCTTTGGCGAGTGGAACGTTATCAGG
GATAAGTGGAATGCCGAGTACGATGATATTCATCTCAAGAAGAAAGCCGTGGTTACAGAGA
AATACGAGGATGATAGACGCAAGAGCTTTAAGAAGATTGGTAGCTTCTCTCTCGAACAGCT
GCAaGAGTACGCCGACGCTGACCTGTCAGTCGTGGAGAAACTCAAGGAGATCATAATCCAG
AAGGTGGATGAAATCTACAAAGTGTATGGAAGCTCTGAGAAACTCTTCGATGCAGACTTTG
TTCTGGAGAAGAGTCTGAAGAAGAACGACGCAGTGGTTGCTATCATGAAGGACCTGCTGG
ATTCTGTTAAGTCTTTCGAGAATTACATTAAGGCATTCTTTGGTGAAGGGAAGGAGACAAAT
AGGGACGAGAGCTTCTATGGCGACTTTGTTCTGGCCTACGACATCCTCCTCAAGGTTGACC
ACATCTATGACGCTATACGGAATTACGTTACCCAGAAGCCCTATAGCAAAGACAAGTTCAAG
CTGTATTTCCAGAATCCACAGTTTATGGGTGGGTGGGATAAAGACAAAGAAACAGATTACA
GGGCCACTATCCTGCGGTACGGCAGCAAATACTATCTGGCTATCATGGATAAGAAGTACGCC
AAATGCCTCCAGAAGATCGACAAGGACGACGTGAACGGTAACTACGAGAAGATCAATTAC
AAGCTCCTGCCAGGACCTAACAAGATGCTGCCCAAGGTGTTCTTCTCCAAGAAATGGATGG
CCTACTATAACCCAAGCGAGGACATTCAGAAGATATACAAGAATGGGACATTCAAGAAGGG
CGATATGTTCAACCTCAACGACTGCCACAAGCTGATTGATTTCTTCAAGGATAGCATTTCTC
GCTATCCCAAGTGGTCTAATGCATACGATTTCAACTTCAGCGAGACTGAGAAGTACAAAGA
CATCGCTGGCTTCTACCGGGAGGTGGAAGAGCAAGGCTATAAGGTGTCATTCGAATCCGCT
TCTAAGAAGGAAGTGGATAAGCTCGTGGAAGAGGGTAAGCTGTACATGTTCCAGATATACA
ACAAAGACTTCAGCGATAAGAGCCACGGCACTCCAAACCTCCATACTATGTATTTCAAGCT
GCTGTTTGACGAGAACAACCACGGACAGATTAGGCTGTCAGGAGGCGCAGAACTCTTCAT
GCGCAGAGCTTCACTGAAGAAGGAGGAACTCGTTGTCCACCCAGCCAATAGCCCTATAGCC
AATAAGAATCCAGACAATCCTAAGAAAACCACTACTCTGTCTTACGATGTGTATAAGGATAA
GAGATTCTCTGAAGATCAGTACGAACTGCACATACCCATTGCCATTAACAAGTGCCCTAAGA
ACATCTTCAAGATTAACACAGAGGTTAGAGTGCTCCTGAAACACGACGATAACCCTTATGTT
ATAGGCATTGCTCGCGGAGAGAGAAACCTGCTGTACATCGTCGTGGTGGACGGCAAAGGC
AACATCGTGGAACAGTACAGTCTCAATGAAATCATTAACAATTTCAACGGAATCCGCATTAA
GACCGACTACCATTCTCTCCTCGACAAGAAGGAGAAAGAAAGGTTCGAAGCAAGACAGAA
TTGGACAAGTATAGAGAATATCAAAGAACTGAAGGCTGGGTACATCTCTCAGGTTGTGCAC
AAGATATGTGAGCTGGTGGAGAAGTACGACGCTGTTATCGCCCTCGCGGACCTGAATAGCG

GCTTCAAGAACTCCAGGGTGAAGGTGGAGAAGCAGGTGTATCAGAAGTTCGAGAAGATGC
TGATCGACAAGCTCAACTATATGGTGGACAAGAAATCCAATCCTTGCGCTACTGGTGGAGC
CCTGAAGGGCTATCAAATCACCAATAAGTTCGAATCTTTCAAGTCTATGAGCACCCAGAATG
GCTTCATCTTCTACATACCCGCATGGCTGACATCCAAGATTGATCCCTCTACCGGATTTGTTA
ATCTGCTCAAGACTAAGTACACCTCTATTGCTGACTCAAAGAAGTTCATATCATCATTTGACC
GCATCATGTACGTGCCAGAAGAGGACCTGTTCGAGTTTGCCCTGGATTACAAGAATTTCTCT
CGGACTGACGCCGACTACATCAAGAAGTGGAAGCTCTACTCTTATGGTAATCGGATTCGCAT
ATTCCGCAATCCCAAGAAGAATAACGTGTTCGATTGGGAGGAAGTTTGCCTCACCAGCGCT
TACAAGGAGCTGTTCAATAAGTATGGGATTAACTACCAGCAGGGCGACATAAGAGCCCTGC
TGTGCGAACAATCTGATAAGGCATTCTATTCCTCTTTCATGGCACTGATGTCACTGATGCTGC
AAATGCGCAATTCCATCACCGGAAGAACAGACGTGGCCTTTCTGATCTCTCCTGTCAAGAA
CTCAGATGGCATCTTCTACGATTCCCGCAACTATGAAGCACAGGAGAATGCTATCCTGCCTA
AGAATGCCGATGCAAATGGAGCCTATAACATCGCCAGAAAGGTCCTCTGGGCCATAGGACA
ATTCAAGAAAGCTGAAGATGAGAAGCTGGACAAGGTGAAGATCGCCATTTCAAACAAAGA
GTGGCTCGAATATGCTCAGACCTCAGTGAAGCATGGATCACCCAAGAAGAAGAGGAAAGT
CTCTGGTGGTTCTACTAATCTGTCAGATATTATTGAAAAGGAAACCGGTAAGCAACTGGTTA
TCCAGGAATCCATCCTCATGCTCCCAGAGGAGGTGGAAGAAGTCATTGGGAACAAGCCGG
AAAGCGATATACTCGTGCACACCGCCTACGACGAGAGCACCGACGAGAATGTCATGCTTCT
GACTAGCGACGCCCCTGAATACAAGCCTTGGGCTCTGGTCATACAGGATAGCAACGGTGAG
AACAAGATTAAGATGCTCTCTGGTGGTTCTCCCAAGAAGAAGAGGAAAGTCTAACCGGTC
ATCATCACCATCACCATTGAGTTTAAACCCGCTGATCAGCCTCTCGAGTAAGGATCTCCAGG
CATCAAATAAAACGAAAGGCTCAGTCGAAAGACTGGGCCTTTCGTTTTATCTGTTGTTTGTC
GGTGAACGCTCTCTACTAGAGTCACACTGGCTCACCTTCGGGTGGGCCTTTCTGCGTTTATA
CCTAGCTCTAGAAATTCCGTCGCAAAAAACCCCGCCCCTGACAGGGCGGGGTTTTTTCGCA
GTCATGCTAGCCTGTGTGAAATTGTTATCCGCTCTGAACCTGCAaGAGGCATCAAATAAAAC
GAAAGGCTCAGTCGAAAGACTGGGCCTTTCGTTTTATCTGTTGTTTGTCGGTGAACGCTCTC
CTGAGTAGGACAAATCCGCCGCCCTAGACCTAGGCGTTCGGCTGCGGCGAGCGGTATCAGC
TCACTCAAAGGCGGTAATACGGTTATCCACAGAATCAGGGGATAACGCAGGAAAGAACATG
TGAGCAAAAGGCCAGCAAAAGGCCAGGAACCGTAAAAAGGCCGCGTTGCTGGCGTTTTTC
CATAGGCTCCGCCCCCCTGACGAGCATCACAAAAATCGACGCTCAAGTCAGAGGTGGCGA
AACCCGACAGGACTATAAAGATACCAGGCGTTTCCCCCTGGAAGCTCCCTCGTGCGCTCTC
CTGTTCCGACCCTGCCGCTTACCGGATACCTGTCCGCCTTTCTCCCTTCGGGAAGCGTGGCG
CTTTCTCAATGCTCACGCTGTAGGTATCTCAGTTCGGTGTAGGTCGTTCGCTCCAAGCTGGG
CTGTGTGCACGAACCCCCCGTTCAGCCCGACCGCTGCGCCTTATCCGGTAACTATCGTCTTG
AGTCCAACCCGGTAAGACACGACTTATCGCCACTGGCAGCAGCCACTGGTAACAGGATTAG
CAGAGCGAGGTATGTAGGCGGTGCTACAGAGTTCTTGAAGTGGTGGCCTAACTACGGCTAC
ACTAGAAGGACAGTATTTGGTATCTGCGCTCTGCTGAAGCCAGTTACCTTCGGAAAAAGAG
TTGGTAGCTCTTGATCCGGCAAACAAACCACCGCTGGTAGCGGTGGTTTTTTTGTTTGCAA
GCAGCAGATTACGCGCAGAAAAAAAGGATCTCAAGAAGATCCTTTGATCTTTTCTACGGGG
TCTGACGCCCGGAATTCAGGCACGAACCCAGTTGACATAAGCCTGTTCGGTTCGTAAACTG
TAATGCAAGTAGCGTATGCGCTCACGCAACTGGTCCAGAACCTTGACCGAACGCAGCGGTG
GTAACGGCGCAGTGGCGGTTTTCATGGCTTGTTATGACTGTTTTTTTGTACAGTCTATGCCTC
GGGCATCCAAGCAGCAAGCGCGTTACGCCGTGGGTCGATGTTTGATGTTATGGAGCAGCAA
CGATGTTACGCAGCAGCAACGATGTTACGCAGCAGGGCAGTCGCCCTAAAACAAAGTTAG

GTGGCTCAAGTATGGGCATCATTCGCACATGTAGGCTCGGCCCTGACCAAGTCAAATCCATG
CGGGCTGCTCTTGATCTTTTCGGTCGTGAGTTCGGAGACGTAGCCACCTACTCCCAACATCA
GCCGGACTCCGATTACCTCGGGAACTTGCTCCGTAGTAAGACATTCATCGCGCTTGCTGCCT
TCGACCAAGAAGCGGTTGTTGGCGCTCTCGCGGCTTACGTTCTGCCCAGGTTTGAGCAGCC
GCGTAGTGAGATCTATATCTATGATCTCGCAGTCTCCGGCGAGCACCGGAGGCAGGGCATTG
CCACCGCGCTCATCAATCTCCTCAAGCATGAGGCCAACGCGCTTGGTGCTTATGTGATCTAC
GTGCAAGCAGATTACGGTGACGATCCCGCAGTGGCTCTCTATACAAAGTTGGGCATACGGG
AAGAAGTGATGCACTTTGATATCGACCCAAGTACCGCCACCTAACAATTCGTTCAAGCCGA
GATCGGCTTCCCGGCCGCGGAGTTGTTCGGTAAATTGTCACAACGCCGCCAGGTGGCACTT
TTCGGGGAAATGTGCGCGCCCGCGTTCCTGCTGGCGCTGGGCCTGTTTCTGGCGCTGGACT
TCCCGCTGTTCCGTCAGCAGCTTTTCGCCCACGGCCTTGATGATCGCGGCGGCCTTGGCCTG
CATATCCCGATTCAACGGCCCCAGGGCGTCCAGAACGGGCTTCAGGCGCTCCCGAAGATCT
CGGGCCGTCTCTTGGGCTTGATCGGCCTTCTTGCGCATCTCACGCGCTCCTGCGGCGGCCTG
TAGGGCAGGCTCATACCCCTGCCGAACCGCTTTTGTCAGCCGGTCGGCCACGGCTTCCGGC
GTCTCAACGCGCTTTGAGATTCCCAGCTTTTCGGCCAATCCCTGCGGTGCATAGGCGCGTG
GCTCGACCGCTTGCGGGCTGATGGTGACGTGGCCCACTGGTGGCCGCTCCAGGGCCTCGTA
GAACGCCTGAATGCGCGTGTGACGTGCCTTGCTGCCCTCGATGCCCCGTTGCAGCCCTAGA
TCGGCCACAGCGGCCGCAAACGTGGTCTGGTCGCGGGTCATCTGCGCTTTGTTGCCGATGA
ACTCCTTGGCCGACAGCCTGCCGTCCTGCGTCAGCGGCACCACGAACGCGGTCATGTGCG
GGCTGGTTTCGTCACGGTGGATGCTGGCCGTCACGATGCGATCCGCCCCGTACTTGTCCGC
CAGCCACTTGTGCGCCTTCTCGAAGAACGCCGCCTGCTGTTCTTGGCTGGCCGACTTCCAC
CATTCCGGGCTGGCCGTCATGACGTACTCGACCGCCAACACAGCGTCCTTGCGCCGCTTCT
CTGGCAGCAACTCGCGCAGTCGGCCCATCGCTTCATCGGTGCTGCTGGCCGCCCAGTGCTC
GTTCTCTGGCGTCCTGCTGGCGTCAGCGTTGGGCGTCTCGCGCTCGCGGTAGGCGTGCTTG
AGACTGGCCGCCACGTTGCCCATTTTCGCCAGCTTCTTGCATCGCATGATCGCGTATGCCGC
CATGCCTGCCCCTCCCTTTTGGTGTCCAACCGGCTCGACGGGGGCAGCGCAAGGCGGTGCC
TCCGGCGGGCCACTCAATGCTTGAGTATACTCACTAGACTTTGCTTCGCAAAGTCGTGACC
GCCTACGGCGGCTGCGGCGCCCTACGGGCTTGCTCTCCGGGCTTCGCCCTGCGCGGTCGCT
GCGCTCCCTTGCCAGCCCGTGGATATGTGGACGATGGCCGCGAGCGGCCACCGGCTGGCTC
GCTTCGCTCGGCCCGTGGACAACCCTGCTGGACAAGCTGATGGACAGGCTGCGCCTGCCC
ACGAGCTTGACCACAGGGATTGCCCACCGGCTACCCAGCCTTCGACCACATACCCACCGGC
TCCAACTGCGCGGCCTGCGGCCTTGCCCCATCAATTTTTTTAATTTTCTCTGGGGAAAAGCC
TCCGGCCTGCGGCCTGCGCGCTTCGCTTGCCGGTTGGACACCAAGTGGAAGGCGGGTCAA
GGCTCGCGCAGCGACCGCGCAGCGGCTTGGCCTTGACGCGCCTGGAACGACCCAAGCCTA
TGCGAGTGGGGGCAGTCGAAGGCGAAGCCCGCCCGCCTGCCCCCCGAGCCTCACGGCGGC
GAGTGCGGGGGTTCCAAGGGGGCAGCGCCACCTTGGGCAAGGCCGAAGGCCGCGCAGTC
GATCAACAAGCCCCGGAGGGGCCACTTTTTGCCGGAGGGGGAGCCGCGCCGAAGGCGTGG
GGGAACCCCGCAGGGGTGCCCTTCTTTGGGCACCAAAGAACTAGATATAGGGCGAAATGC
GAAAGACTTAAAAATCAACAACTTAAAAAAGGGGGGTACGCAACAGCTCATTGCGGCACC
CCCCGCAATAGCTCATTGCGTAGGTTAAAGAAAATCTGTAATTGACTGCCACTTTTACGCAA
CGCATAATTGTTGTCGCGCTGCCGAAAAGTTGCAGCTGATTGCGCATGGTGCCGCAACCGT
GCGGCACCCTACCGCATGGAGATAAGCATGGCCACGCAGTCCAGAGAAATCGGCATTCAAG
CCAAGAACAAGCCCGGTCACTGGGTGCAAACGGAACGCAAAGCGCATGAGGCGTGGGCC
GGGCTTATTGCGAGGAAACCCACGGCGGCAATGCTGCTGCATCACCTCGTGGCGCAGATGG

GCCACCAGAACGCCGTGGTGGTCAGCCAGAAGACACTTTCCAAGCTCATCGGACGTTCTTT
GCGGACGGTCCAATACGCAGTCAAGGACTTGGTGGCCGAGCGCTGGATCTCCGTCGTGAA
GCTCAACGGCCCCGGCACCGTGTCGGCCTACGTGGTCAATGACCGCGTGGCGTGGGGCCA
GCCCCGCGACCAGTTGCGCCTGTCGGTGTTCAGTGCCGCCGTGGTGGTTGATCACGACGAC
CAGGACGAATCGCTGTTGGGGCATGGCGACCTGCGCCGCATCCCGACCCTGTATCCGGGCG
AGCAGCAACTACCGACCGGCCCCGGCGAGGAGCCGCCCAGCCAGCCCGGCATTCCGGGCA
TGGAACCAGACCTGCCAGCCTTGACCGAAACGGAGGAATGGGAACGGCGCGGGCAGCAG
CGCCTGCCGATGCCCGATGAGCCGTGTTTTCTGGACGATGGCGAGCCGTTGGAGCCGCCGA
CACGGGTCACGCTGCCGCGCCGGTAGCACTTGGGTTGCGCAGCAACCCGTAAGTGCGCTG
TTCCAGACTATCGGCTGTAGCCGCCTCGCCGCCCTATACCTTGTCTGCCTCCCCGCGTTGCG
TCGCGGTGCATGGAGCCGGGCCACCTCGACCTGAATGGAAGCCGGCGGCACCTCGCTAAC
GGATTCACCGTTTTTATCAGGCTCTGGGAGGCAGAATAAATGATCATATCGTCAATTATTACC
TCCACGGGGAGAGCCTGAGCAAACTGGCCTCAGGCATTTGAGAAGCAC

Supplementary Notes 2.

Preparation and transformation conditions of *Psa* chemically competent cell

Methods

PSA.AH.01 was taken out from -70 °C and incubated with King's medium(containing 50μg mL^{-1} Rifampicin) at 30°C for 24h. PSA.AH.01 bacterial solution was streaked on the King's plate medium(containing 50μg mL^{-1} Rifampicin) and cultured at 30°C for 24h. Single colony of PSA.AH.01 was pick into 10mL liquid King's medium (containing 50μg mL^{-1} Rifampicin) and cultured with 200rpm min^{-1} for 14h at 30°C. 2mL single colony bacterial solution was added into the 200mL fresh liquid King's medium and incubated with 200rpm min^{-1} for 5~8h at 30°C until the OD600 of bacterial solution reached about 0.5. Then the bacterial solution were tranfered into 50mL centrifuge tube and ice-bath for 20 minutes. The bacterial solution were centrifuged with 7000 × g for 10 minutes at 4°C, bacterial cells were collected and washed with distilled water 3 times. The bacterial cells were added different concentrations $CaCl_2$ solution(25mmol/L, 50mmol/L, 75mmol/L, 100mmol/L) and mixed well with ice-bath for 1h, then centrifuged with 7000 × g for 10 minutes at 4°C. The supernatant were discarded and the bacterial cells were added 0.7mL $CaCl_2$ and 0.3mL 50% glycerol(glycerol:H_2O was 1:1), mixed and dispensed into each centrifuge tubes with 100μL. The centrifuge tubes with bacterial cells were immediately freezed in liquid nitrogen for 5 min and stored at -70°C.

The competent cells treated with different concentrations of $CaCl_2$ were thawed on ice, added 2μL of pEASY-Blunt simple vector, mixed well and bathed on ice for 1h. Then the competent cells with vector were heat shock in a 42°C water bath for 1.5min, 3min, 4.5min, and 6min respectively, and then quickly transfered to an ice-water bath for 5min. The competent cells were added 700 μL antibiotic-free liquid King's medium, mixed well and recovered on a shaker with 200rpm min^{-1} at 30°C for 1 h, 2 h, 3 h, and 4 h, respectively. It was centrifuged at 3500 rpm min^{-1} for 5 min, removed the supernatant in the ultra-clean workbench, retained about 200 μL of solution, mixed well and spreaded evenly on King's solid medium(containing 50 μg/mL Kana) at 30°C, cultured for 24~36 h.

Orthogonal experiments with 3 factors and 4 levels were carried out to study the effects of different $CaCl_2$ concentrations, heat shock time, and recovery time on the preparation and transformation conditions of *Psa* competent cells, as shown in Supplementary Table S2.

Supplementary Notes 3

Complete sequence of pEASY-sgRNA-Trc vector

AGCGCCCAATACGCAAACCGCCTCTCCCCGCGCGTTGGCCGATTCATTAATGCAGCTGGCA
CGACAGGTTTCCCGACTGGAAAGCGGGCAGTGAGCGCAACGCAATTAATGTGAGTTAGCT
CACTCATTAGGCACCCCAGGCTTTACACTTTATGCTTCCGGCTCGTATGTTGTGTGGAATTGT
GAGCGGATAACAATTTCACACAGGAAACAGCTATGACCATGATTACGCCAAGCTGCCCTTG
TTTTAGAGCTAGGCCAACATGAGGATCACCCATGTCTGCAGGGCCTAGCAAGTTAAAATAA
GGCTAGTCCGTTATCAACTTGGCCAACATGAGGATCACCCATGTCTGCAGGGCCAAGTGGC
ACCGAGTCGGTGCTTTTTCTGCAGGAGGCATTTGACAATTAATCATCCGGCTCGTATAATGA
AGGGCAGCTTCAATTCGCCCTATAGTGAGTCGTATTACAATTCACTGGCCGTCGTTTTACAA
CGTCGTGACTGGGAAAACCCTGGCGTTACCCAACTTAATCGCCTTGCAGCACATCCCCCTTT
CGCCAGCTGGCGTAATAGCGAAGAGGCCCGCACCGATCGCCCTTCCCAACAGTTGCGCAGC
CTGAATGGCGAATGGACGCGCCCTGTAGCGGCGCATTAAGCGCGGCGGGTGTGGTGGTTAC
GCGCAGCGTGACCGCTACACTTGCCAGCGCCCTAGCGCCCGCTCCTTTCGCTTTCTTCCCTT
CCTTTCTCGCCACGTTCGCCGGCTTTCCCCGTCAAGCTCTAAATCGGGGGCTCCCTTTAGGG
TTCCGATTTAGTGCTTTACGGCACCTCGACCCCAAAAAACTTGATTAGGGTGATGGTTCACG
TAGTGGGCCATCGCCCTGATAGACGGTTTTTCGCCCTTTGACGTTGGAGTCCACGTTCTTTA
ATAGTGGACTCTTGTTCCAAACTGGAACAACACTCAACCCTATCTCGGTCTATTCTTTTGATT
TATAAGGGATTTTGCCGATTTCGGCCTATTGGTTAAAAAATGAGCTGATTTAACAAAAATTTA
ACGCGAATTTTAACAAAATTCAGGGCGCAAGGGCTGCTAAAGGAAGCGGAACACGTAGAA
AGCCAGTCCGCAGAAACGGTGCTGACCCCGGATGAATGTCAGCTACTGGGCTATCTGGACA
AGGGAAAACGCAAGCGCAAAGAGAAAGCAGGTAGCTTGCAGTGGGCTTACATGGCGATAG
CTAGACTGGGCGGTTTTATGGACAGCAAGCGAACCGGAATTGCCAGCTGGGGCGCCCTCTG
GTAAGGTTGGGAAGCCCTGCAAAGTAAACTGGATGGCTTTCTTGCCGCCAAGGATCTGATG
GCGCAGGGGATCAAGATCTGATCAAGAGACAGGATGAGGATCGTTTCGCATGATTGAACAA
GATGGATTGCACGCAGGTTCTCCGGCCGCTTGGGTGGAGAGGCTATTCGGCTATGACTGGG
CACAACAGACAATCGGCTGCTCTGATGCCGCCGTGTTCCGGCTGTCAGCGCAGGGGCGCCC
GGTTCTTTTTGTCAAGACCGACCTGTCCGGTGCCCTGAATGAACTGCAGGACGAGGCAGCG
CGGCTATCGTGGCTGGCCACGACGGGCGTTCCTTGCGCAGCTGTGCTCGACGTTGTCACTG
AAGCGGGAAGGGACTGGCTGCTATTGGGCGAAGTGCCGGGGCAGGATCTCCTGTCATCCC
ACCTTGCTCCTGCCGAGAAAGTATCCATCATGGCTGATGCAATGCGGCGGCTGCATACGCTT
GATCCGGCTACCTGCCCATTCGACCACCAAGCGAAACATCGCATCGAGCGAGCACGTACTC
GGATGGAAGCCGGTCTTGTCGATCAGGATGATCTGGACGAAGAGCATCAGGGGCTCGCGCC
AGCCGAACTGTTCGCCAGGCTCAAGGCGCGCATGCCCGACGGCGAGGATCTCGTCGTGAC
CCACGGCGATGCCTGCTTGCCGAATATCATGGTGGAAAATGGCCGCTTTTCTGGATTCATCG
ACTGTGGCCGGCTGGGTGTGGCGGACCGCTATCAGGACATAGCGTTGGCTACCCGTGATATT
GCTGAAGAGCTTGGCGGCGAATGGGCTGACCGCTTCCTCGTGCTTTACGGTATCGCCGCTC
CCGATTCGCAGCGCATCGCCTTCTATCGCCTTCTTGACGAGTTCTTCTGAATTGAAAAAGGA
AGAGTATGAGTATTCAACATTTCCGTGTCGCCCTTATTCCCTTTTTTGCGGCATTTTGCCTTC
CTGTTTTTGCTCACCCAGAAACGCTGGTGAAAGTAAAAGATGCTGAAGATCAGTTGGGTGC
ACGAGTGGGTTACATCGAACTGGATCTCAACAGCGGTAAGATCCTTGAGAGTTTTCGCCCC
GAAGAACGTTTTCCAATGATGAGCACTTTTAAAGTTCTGCTATGTGGCGCGGTATTATCCCG
TATTGACGCCGGGCAAGAGCAACTCGGTCGCCGCATACACTATTCTCAGAATGACTTGGTT

GAGTACTCACCAGTCACAGAAAAGCATCTTACGGATGGCATGACAGTAAGAGAATTATGCA
GTGCTGCCATAACCATGAGTGATAACACTGCGGCCAACTTACTTCTGACAACGATCGGAGG
ACCGAAGGAGCTAACCGCTTTTTTGCACAACATGGGGGATCATGTAACTCGCCTTGATCGTT
GGGAACCGGAGCTGAATGAAGCCATACCAAACGACGAGCGTGACACCACGATGCCTGTAG
CAATGGCAACAACGTTGCGCAAACTATTAACTGGCGAACTACTTACTCTAGCTTCCCGGCA
ACAATTAATAGACTGGATGGAGGCGGATAAAGTTGCAGGACCACTTCTGCGCTCGGCCCTT
CCGGCTGGCTGGTTTATTGCTGATAAATCTGGAGCCGGTGAGCGTGGGTCTCGCGGTATCAT
TGCAGCACTGGGGCCAGATGGTAAGCCCTCCCGTATCGTAGTTATCTACACGACGGGGAGT
CAGGCAACTATGGATGAACGAAATAGACAGATCGCTGAGATAGGTGCCTCACTGATTAAGC
ATTGGTAACTGTCAGACCAAGTTTACTCATATATACTTTAGATTGATTTAAAACTTCATTTTTA
ATTTAAAAGGATCTAGGTGAAGATCCTTTTTGATAATCTCATGACCAAAATCCCTTAACGTG
AGTTTTCGTTCCACTGAGCGTCAGACCCCGTAGAAAAGATCAAAGGATCTTCTTGAGATCC
TTTTTTTCTGCGCGTAATCTGCTGCTTGCAAACAAAAAAACCACCGCTACCAGCGGTGGTTT
GTTTGCCGGATCAAGAGCTACCAACTCTTTTTCCGAAGGTAACTGGCTTCAGCAGAGCGCA
GATACCAAATACTGTTCTTCTAGTGTAGCCGTAGTTAGGCCACCACTTCAAGAACTCTGTAG
CACCGCCTACATACCTCGCTCTGCTAATCCTGTTACCAGTGGCTGCTGCCAGTGGCGATAAG
TCGTGTCTTACCGGGTTGGACTCAAGACGATAGTTACCGGATAAGGCGCAGCGGTCGGGCT
GAACGGGGGGTTCGTGCACACAGCCCAGCTTGGAGCGAACGACCTACACCGAACTGAGAT
ACCTACAGCGTGAGCTATGAGAAAGCGCCACGCTTCCCGAAGGGAGAAAGGCGGACAGGT
ATCCGGTAAGCGGCAGGGTCGGAACAGGAGAGCGCACGAGGGAGCTTCCAGGGGGAAAC
GCCTGGTATCTTTATAGTCCTGTCGGGTTTCGCCACCTCTGACTTGAGCGTCGATTTTTGTGA
TGCTCGTCAGGGGGGCGGAGCCTATGGAAAAACGCCAGCAACGCGGCCTTTTTACGGTTCC
TGGCCTTTTGCTGGCCTTTTGCTCACATGTTCTTTCCTGCGTTATCCCCTGATTCTGTGGATA
ACCGTATTACCGCCTTTGAGTGAGCTGATACCGCTCGCCGCAGCCGAACGACCGAGCGCAG
CGAGTCAGTGAGCGAGGAAGCGGAAG

Supplementary Notes 4

Screening code of knockout target in Psa genome with dCas9-BE3

```
#import Lib
import argparse

BASE_DICT={
	'A':'T',
	'T':'A',
	'G':'C',
	'C':'G',
	'N':'N'
}
def getSeq(infile):
	with open(infile,'r') as fr:
		seqDict={}
		for seq in fr.readlines():
			if seq[0] == '>':
				seqName=seq.strip()
				seqDict[seqName]=''
			else:
				seqDict[seqName]+=seq.strip()

	return seqDict

def getReverseSeq(seq):
	newSeq=''
	for base in seq[::-1]:
		newSeq+=BASE_DICT[base]
	return newSeq

def
getFragmentFromSeq(seqStr,strand='+',fragment='GG',fragmentHash={'CAG,CAA,C
GA'},\
	fragmentLength=2,fragmentHashLength=3,start=21,front=12,end=18):
	fragmentList=[]
	resultList=[]
	count=1
	if strand=='-':
		seqStr=getReverseSeq(seqStr)
		fragmentHash={'CCA'}
	for idxi in range(start,len(seqStr)-fragmentLength+1,1):
		tempFragment=seqStr[idxi:idxi+fragmentLength]
```

```
            if tempFragment==fragment:
                for idxj in range(idxi-end,idxi-front+1,1):
                    tempFragmentHash=seqStr[idxj:idxj+fragmentHashLength]
                    if tempFragmentHash in fragmentHash:
                        idxk=idxj
                        if strand=='-':
                            idxk=len(seqStr)-idxj
                        if (not(idxk % 3)) and (seqStr[idxj-1] != 'G'):
                            specialSeq= seqStr[idxi-21:idxi-1]

result=(count,strand,idxi+1,idxj+1,(idxi+1)/len(seqStr),seqStr[idxi-21:idxi-1])
                            if specialSeq not in fragmentList:
                                fragmentList.append(specialSeq)
                                resultList.append(result)
                                count+=1
    return resultList

def
getFragmentFromSeqList(seqStrDict,fragment='GG',fragmentHash={'CAG,CAA,CG
A'},\
    fragmentLength=2,fragmentHashLength=3,start=21,front=12,end=18):

    '''
        forward and    reverse
    '''
    outReDict={}
    for seq in seqStrDict:
        seqStr=seqStrDict[seq]
        seqReList=getFragmentFromSeq(seqStr,'+',fragment,fragmentHash,\
            fragmentLength,fragmentHashLength,start,front,end)
        rseqReList=getFragmentFromSeq(seqStr,'-',fragment,fragmentHash,\
            fragmentLength,fragmentHashLength,start,front,end)
        seqReList.extend(rseqReList)
        if seq not in outReDict:
            outReDict[seq]=seqReList
    return outReDict

def fragWrite(outReDict,outputfile):
    if outReDict:
        with open(outputfile,'w') as fw:
            for seqName in outReDict:
                fw.write(seqName+'\n')
                fw.write('ID\tStrand\tGG\tC\tlocal\tSequence\n')
                for fragment in outReDict[seqName]:
```

```
fw.write('seq%d\t%s\t%d\t%d\t%0.2f\t%s\n'%(fragment[0],fragment[1],\
                            fragment[2],fragment[3],fragment[4],fragment[5]))
                    fw.write('\n')
    else:
        print("can't find any seqName!")

def initParse():
    parser=argparse.ArgumentParser(description="this script is used to find special
sequence")
    parser.add_argument("-i",dest="inputfile",help="input sequence file(fasta)")
    parser.add_argument("-o",dest="outputfile",help="output                special
sequence(fasta)")
    args=parser.parse_args()
    return args.inputfile,args.outputfile

def main():
    inputfile,outputfile=initParse()
    seqStrDict=getSeq(inputfile)

outReDict=getFragmentFromSeqList(seqStrDict,'GG',{'CAG','CAA','CGA'},2,3,21,1
2,18)
    fragWrite(outReDict,outputfile)

if __name__ == '__main__':
main()
```

Supplementary Notes 5.

Screening code of knockout target in Psa genome with dCas12a-BE3

```
#import Lib
import argparse

BASE_DICT={
	'A':'T',
	'T':'A',
	'G':'C',
	'C':'G',
	'N':'N'
}
def getSeq(infile):
	with open(infile,'r') as fr:
		seqDict={}
		for seq in fr.readlines():
			if seq[0] == '>':
				seqName=seq.strip()
				seqDict[seqName]=''
			else:
				seqDict[seqName]+=seq.strip()

	return seqDict

def getReverseSeq(seq):
	newSeq=''
	for base in seq[::-1]:
		newSeq+=BASE_DICT[base]
	return newSeq

def
getFragmentFromSeq(seqStr,strand='+',fragment='TTT',fragmentHash={'CAG,CAA,
CGA'},\
	fragmentLength=3,fragmentHashLength=3,start=0,front=11,end=17):
	fragmentList=[]
	resultList=[]
	count=1
	if strand=='-':
		seqStr=getReverseSeq(seqStr)
		fragmentHash={'CCA'}
	for idxi in range(start,len(seqStr)-fragmentLength+1,1):
		tempFragment=seqStr[idxi:idxi+fragmentLength]
```

```
            if tempFragment==fragment:
                for idxj in range(idxi+front,idxi+end+1,1):
                    tempFragmentHash=seqStr[idxj:idxj+fragmentHashLength]
                    if tempFragmentHash in fragmentHash:
                        idxk=idxj
                        if seqStr[idxi+3]=='T':
                            continue
                        if strand=='-':
                            idxk=len(seqStr)-idxj
                        if (not(idxk % 3)) and (seqStr[idxj-1] != 'G'):
                            specialSeq= seqStr[idxi+4:idxi+24]

result=(count,strand,idxi+1,idxj+1,(idxi+1)/len(seqStr),seqStr[idxi+4:idxi+24])
                            if specialSeq not in fragmentList:
                                fragmentList.append(specialSeq)
                                resultList.append(result)
                                count+=1
    return resultList

def
getFragmentFromSeqList(seqStrDict,fragment='GG',fragmentHash={'CAG,CAA,CG
A'},\
    fragmentLength=3,fragmentHashLength=3,start=0,front=11,end=17):

    '''
        forward and    reverse
    '''
    outReDict={}
    for seq in seqStrDict:
        seqStr=seqStrDict[seq]
        seqReList=getFragmentFromSeq(seqStr,'+',fragment,fragmentHash,\
            fragmentLength,fragmentHashLength,start,front,end)
        rseqReList=getFragmentFromSeq(seqStr,'-',fragment,fragmentHash,\
            fragmentLength,fragmentHashLength,start,front,end)
        seqReList.extend(rseqReList)
        if seq not in outReDict:
            outReDict[seq]=seqReList
    return outReDict

def fragWrite(outReDict,outputfile):
    if outReDict:
        with open(outputfile,'w') as fw:
            for seqName in outReDict:
                fw.write(seqName+'\n')
```

```
                fw.write('ID\tStrand\tGG\tC\tlocal\tSequence\n')
                for fragment in outReDict[seqName]:

fw.write('seq%d\t%s\t%d\t%d\t%0.2f\t%s\n'%(fragment[0],fragment[1],\
                        fragment[2],fragment[3],fragment[4],fragment[5]))
                fw.write('\n')
    else:
        print("can't find any seqName!")

def initParse():
    parser=argparse.ArgumentParser(description="this script is used to find special
sequence")
    parser.add_argument("-i",dest="inputfile",help="input sequence file(fasta)")
    parser.add_argument("-o",dest="outputfile",help="output                special
sequence(fasta)")
    args=parser.parse_args()
    return args.inputfile,args.outputfile

def main():
    inputfile,outputfile=initParse()
    seqStrDict=getSeq(inputfile)

outReDict=getFragmentFromSeqList(seqStrDict,'TTT',{'CAG','CAA','CGA'},3,3,0,11
,17)
    fragWrite(outReDict,outputfile)

if __name__ == '__main__':
main()
```
